# Adiponectin Deficiency Promotes Tumor Growth in Mice by Reducing Macrophage Infiltration

**DOI:** 10.1371/journal.pone.0011987

**Published:** 2010-08-05

**Authors:** Yutong Sun, Harvey F. Lodish

**Affiliations:** Whitehead Institute for Biomedical Research, Cambridge, Massachusetts, United States of America; Louisiana State University, United States of America

## Abstract

Adiponectin is an adipocyte-derived plasma protein that has been implicated in regulating angiogenesis, but the role of adiponectin in regulating this process is still controversial. In this study, in order to determine whether adiponectin affects tumor growth and tumor induced vascularization, we implanted B16F10 melanoma and Lewis Lung Carcinoma cells subcutaneously into adiponectin knockout and wild-type control mice, and found that adiponectin deficiency markedly promoted the growth of both tumors. Immunohistochemical analyses indicated that adiponectin deficiency reduced macrophage recruitment to the tumor, but did not affect cancer cell mitosis, apoptosis, or tumor-associated angiogenesis. In addition, treatment with recombinant adiponectin did not affect the proliferation of cultured B16F10 tumor cells. Importantly, the restoration of microphage infiltration at an early stage of tumorigenesis by means of co-injection of B16F10 cells and macrophages reversed the increased tumor growth in adiponectin knockout mice. Thus, we conclude that the enhanced tumor growth observed in adiponectin deficient mice is likely due to the reduction of macrophage infiltration rather than enhanced angiogenesis.

## Introduction

Adiponectin, also named Acrp30, is an adipocyte-derived plasma protein which plays an important role in regulating fat and glucose metabolism. Serum levels of adiponectin are reduced in obese individuals, and inversely correlate with several diseases, including type II diabetes [1], cardiovascular diseases [2] and metabolic syndromes [3], as well as the risk of developing multiple types of cancers [4].

Although adiponectin has been suggested to be one of the regulators of angiogenesis, its precise role in regulating this process is still not clear. One group, employing in vitro cell culture systems and other angiogenesis models, showed that adiponectin can stimulate angiogenesis [5], [6]. In addition, two recent papers [7], [8] demonstrated that adiponectin deficiency reduced primary tumor-induced vascularization, indicating that adiponectin might be pro-angiogenic. However, another study, using different models, suggested that adiponectin is a *negative* regulator of angiogenesis [9], which is also supported by a recent finding [10] that the administration of adenovirus expressing adiponectin suppressed liver tumor growth in nude mice by inhibiting angiogenesis. These discrepancies may be due to the different experimental systems employed in these studies.

In the present study, in order to determine the role of adiponectin in tumor growth and tumor induced angiogenesis, we investigated whether adiponectin deficiency has any effect on the growth rate of tumor cells implanted in syngeneic and immunocompetent mice. We found that the loss of adiponectin significantly promoted tumor growth in vivo, but did not affect tumor-associated vascularization. Mechanistic insights into this phenotype suggest that adiponectin deficiency enhances tumor growth in mice most likely by reducing macrophage infiltration.

## Results

### Adiponectin deficiency enhances primary tumor growth but does not affect metastatic colonization

**Figure 1:**
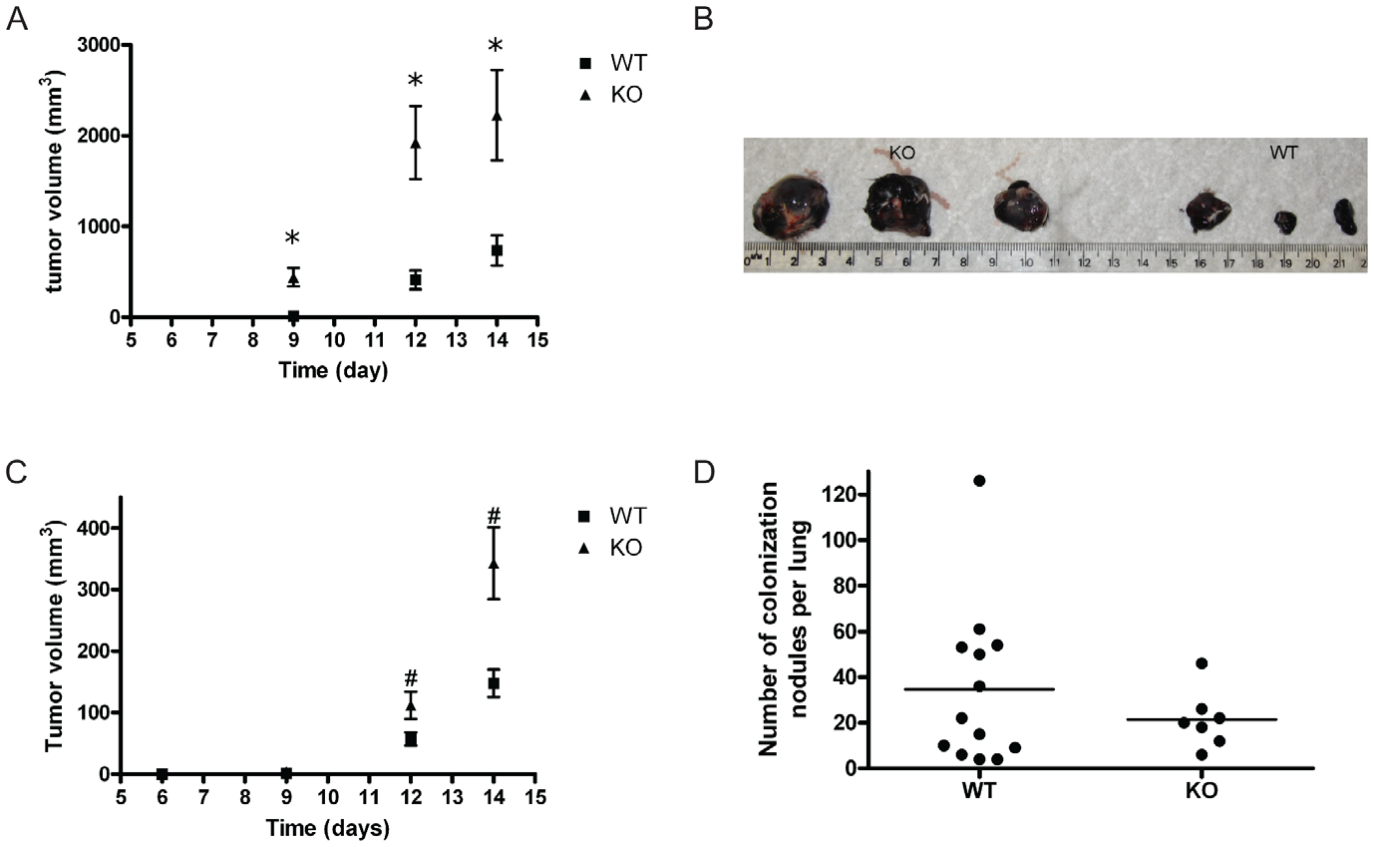
Enhanced tumor growth in adiponectin-deficient mice. (A) Tumor volume of B16F10 melanoma cells in adiponectin knockout (KO) mice and wild-type (WT) control mice as a function of time. (KO, n = 10; WT n = 6. *: p<0.05). (B) Photograph of B16F10 tumors dissected 14 days after the injection (scale bar  = 10 mm). (C) Tumor volume of Lewis Lung Carcinoma (LLC) cells in adiponectin KO mice and WT control mice as a function of time. (KO, n = 8; WT n = 7. #: p<0.05) (D) Total numbers of metastatic nodules in the lung of individual WT or adiponectin KO mice. (KO, n = 7; WT n = 13. p>0.05)

Adiponectin knockout mice in our lab were maintained in a C57BL/6J background and both B16F10 melanoma cells and Lewis Lung Carcinoma (LLC) cells were derived from the same (C57BL/6J) background. In order to determine the role of adiponectin in regulating tumor growth, we implanted B16F10 cells or LLC cells subcutaneously into adiponectin knockout (KO) and control C57BL/6J (WT) mice. As shown in Figures 1A, B and C, B16F10 and LLC tumor cells were able to grow in both adiponectin KO and wild type (WT) mice. Notably, tumor cells implanted in adiponectin KO mice grew more rapidly than those implanted in WT mice; at 14 days post implantation, the average tumor volume in adiponectin KO mice was approximately 3-fold larger than that in control mice (Figures 1A and C, p<0.05).

At 14 days post implantation, the majority of B16F10 and LLC tumors growing in adiponectin knockout mice were over 2 cm in diameter, which met the euthanasia criteria in our animal protocol. Therefore, we terminated these experiments at 2 weeks after tumor cell implantation. No macroscopic lung metastases were observed at this time point (data not shown). To determine whether adiponectin deficiency had any effect on the metastatic ability of B16F10 cells, we injected the B16F10 cells into mice through the tail vein, and did not observe a significant difference in lung colonization between the adiponectin deficient mice and the wild-type control animals, as quantified by counting lung nodules in individual mice (Figure 1D, p>0.05).

Because previous reports have suggested that recombinant adiponectin was able to inhibit the proliferation of cultured breast cancer cell lines (MDA-MB-231 and MCF-7) [11], [12], we investigated whether recombinant adiponectin can also suppress B16F10 cell proliferation in vitro. As shown in supplemental Figure S1, although purified mouse recombinant adiponectin could inhibit MDA-MB-231 cell proliferation at a concentration of 30 µg/ml, the same recombinant protein had no effect on B16F10 cell proliferation in vitro, suggesting that the inhibitory effects of adiponectin on cell proliferation are cell-type dependent and that other mechanisms by which adiponectin deficiency promotes tumor growth in mice are yet to be identified.

### Apoptosis, mitosis and angiogenesis are not affected but macrophage recruitment to the tumor is reduced in the absence of adiponectin

**Figure 2:**
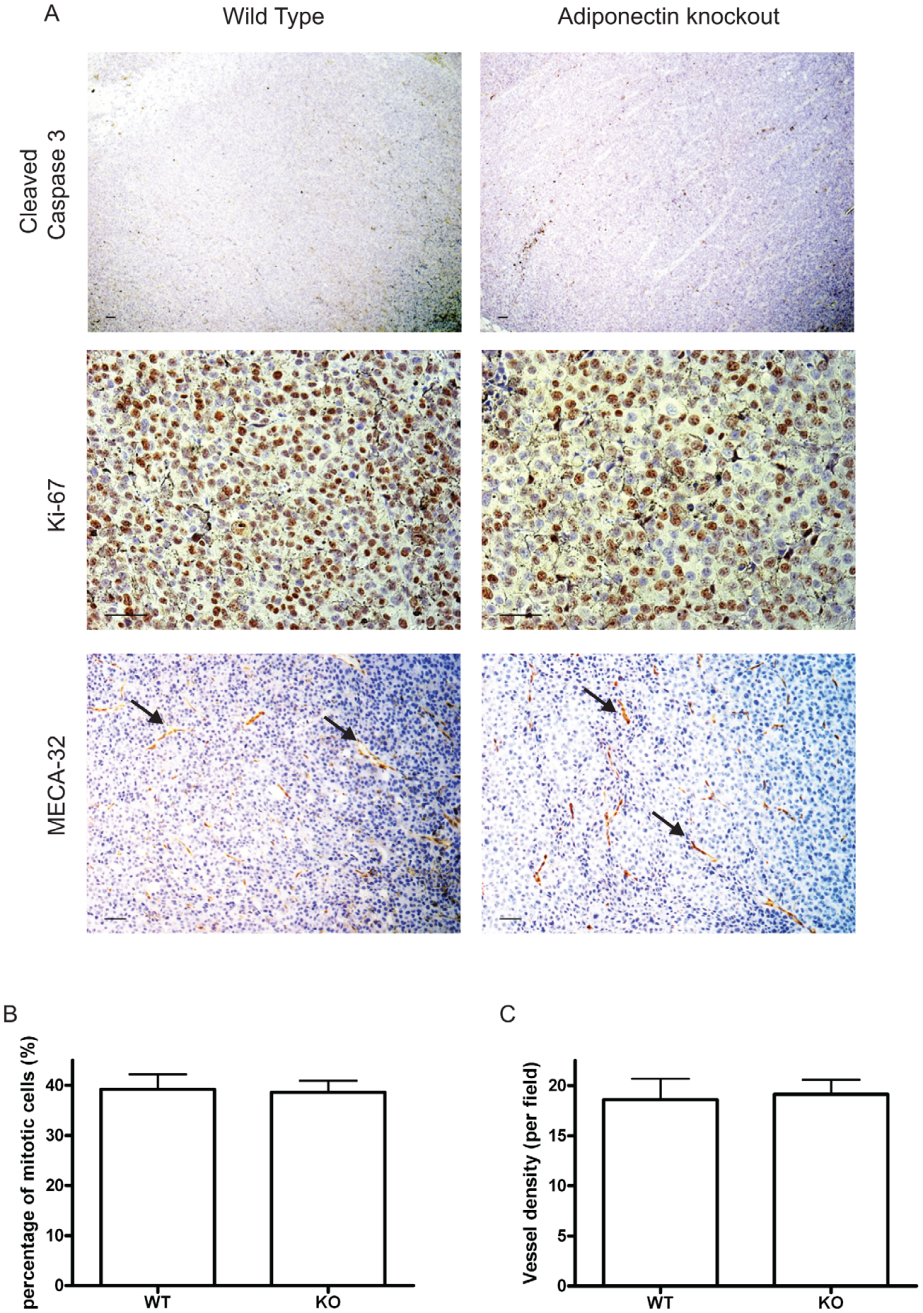
B16F10 tumors from adiponectin knockout (KO) mice display similar apoptosis, mitosis and angiogenesis compared with those from wild type (WT) mice. A. Immunohistochemistry staining of B16F10 tumor sections from wild type and adiponectin knockout mice. Antibodies: cleaved caspase 3, Ki-67 and MECA-32 (vessels are brown and indicated by arrows. scale bar, 50 µm). B. Percentage of mitotic cells does not change in tumors from adiponectin null mice (Fields counted: WT, n = 5; KO, n = 5. p>0.05). C. Adiponectin deficiency does not affect vessel density in transplanted tumors (Fields counted: WT, n = 17; KO, n = 24. p>0.05).

B16F10 and LLC tumor sections were analyzed 14 days after cancer cell implantation. Immunohistochemical (IHC) analysis of cleaved caspase 3 (Figure 2A and supplemental Figure S2A), a marker for apoptotic cells; Ki-67 (Figures 2A, B and supplemental Figures S2A, B), a marker for mitotic cells; and MECA-32 (Figures 2A, C and supplemental Figures S2A, C), an endothelial cell maker, showed that adiponectin deficiency in mice had no effect on tumor cell apoptosis, mitosis, or angiogenesis. Since apoptotic cells were clustered throughout the tumors and unevenly distributed, precise quantification was performed using FACS analysis by annexin V and 7AAD staining (supplemental Figure S3), which indicated no reduction in apoptosis in B16F10 tumors from adiponectin null mice: at day 9, the percentage of apoptotic cells in B16F10 tumors was 8.42±3.62% (n = 6) in wild-type mice and 7.78±3.21% in knock out mice (n = 5, p = 0.77).

**Figure 3:**
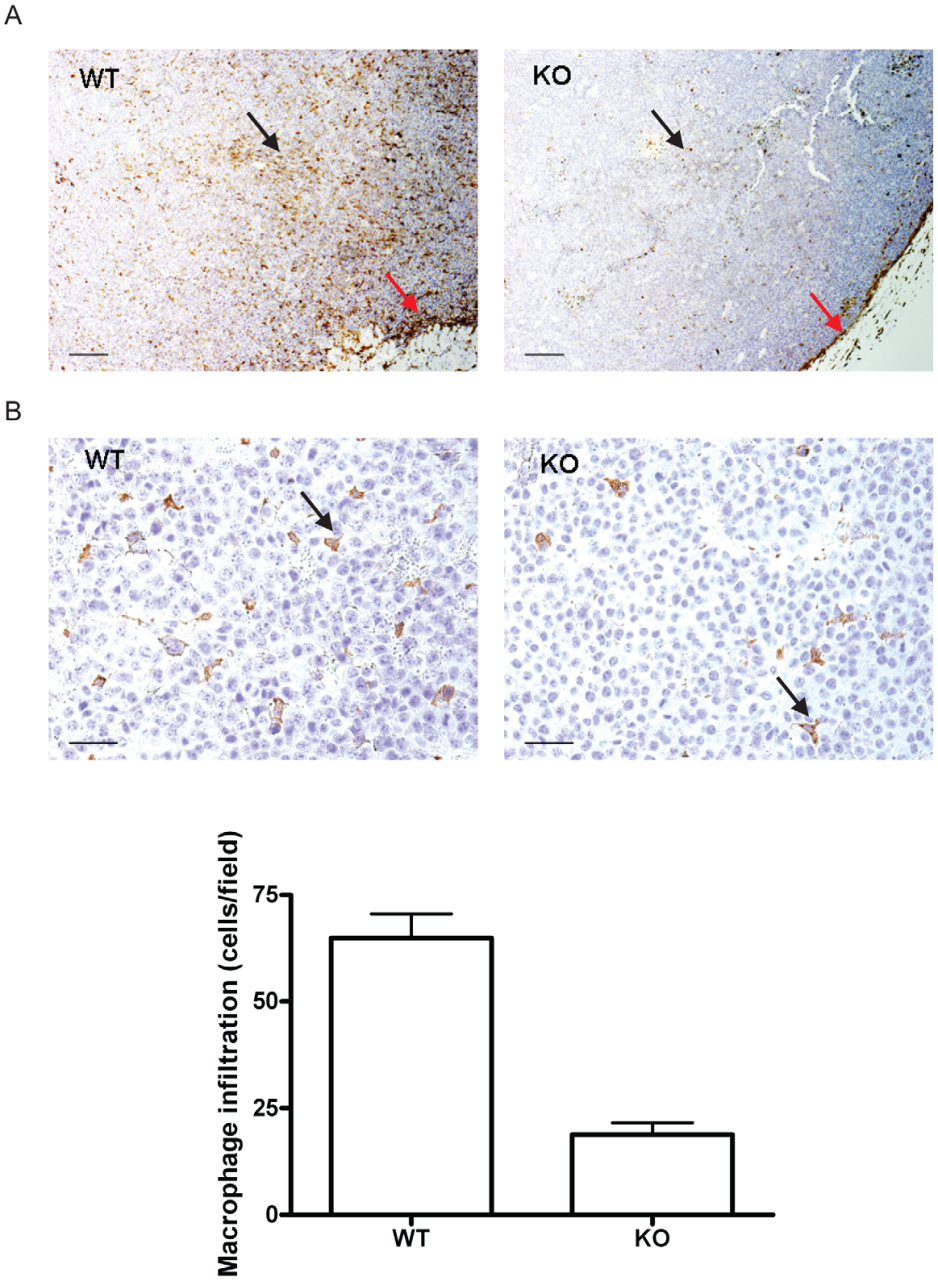
Macrophage infiltration is reduced in B16F10 tumors from adiponectin knockout (KO) mice. A. Immunohistochemistry staining of B16F10 tumor sections from wild type (WT) and adiponectin knockout (KO) mice against an anti-macrophage antibody F4/80. A lower infiltration of macrophages (brown, indicated by black arrows; red arrows indicate the macrophage infiltration on the edge of tumors) was observed in tumors grown in adiponectin null mice. (Scale bar, A: 200 µm, B: 50 µm). B. Microscopic counts indicate that adiponectin deficiency decreases intra-tumor macrophage infiltration (Fields counted: WT, n = 17; KO n = 23; p<0.05).

Since accumulating evidence [13], [14], [15], [16] has demonstrated that adiponectin may modulate macrophage functions, and since macrophages are involved in regulating tumor growth and progression, we decided to examine the degree of macrophage infiltration by immunohistochemical staining with the anti-F4/80 antibody, which recognizes a specific macrophage antigen. To avoid experimenter bias, immunohistochemical analysis and counting were conducted by an independent person blinded to the adiponectin status. As shown in Figure 3A and supplemental Figure S4A, macrophage infiltration was observed within the tumors growing in adiponectin KO and WT mice. Although the distribution of macrophages within each tumor was uneven and more microphage infiltration was observed at the edge of tumors, there was a clear reduction in macrophage recruitment to tumors from adiponectin deficient mice than those from wild-type mice (Figure 3A and supplemental Figure S4A). Microscopic examination revealed that the infiltration of macrophages was reduced by about 3-fold within tumors from adiponectin KO mice (Figure 3B and supplemental Figure S4B, p<0.05). The numbers of circulating B, T cells, granulocytes and monocytes were not significantly altered in adiponectin KO mice (supplemental Figure S5). Therefore, we concluded that the infiltration of macrophages into the tumor was specifically decreased in adiponectin null mice.

### Co-implantation of macrophages with B16F10 cells reverses the observed increase in tumor growth in adiponectin KO mice

Based on these observations, we hypothesized that macrophages might play an inhibitory role in tumor growth in the tumor models used in the present study. Macrophages are considered to be highly phagocytic, essential immune effector cells that are derived from monocytes that leave the blood, enter tissues, and differentiate. Tumor associated macrophages (TAMs) are one of the key regulators that link inflammation and cancer. Accumulating evidence suggests that TAMs display substantial phenotypic heterogeneity and may play a dual role in tumor growth [17]: they can be tumoricidal [18], [19] *at the early stages of tumorigenesis* through macrophage-mediated cytotoxicity [20] and phagocytosis [18]. On the other hand, TAMs can *be educated by the tumor microenvironment* and are capable of producing a number of angiogenic growth factors, cytokines, and proteases, which may promote tumor progression and metastasis [21].

In order to determine whether macrophages can reverse the enhanced tumor growth in adiponectin KO mice, we wished to restore macrophages to these tumors. To this end, we took advantage of a well-established protocol that utilizes sterile eliciting agents to recruit immature macrophages into the mouse peritoneal cavity, resulting in an approximately 10 times increased yield of macrophages [22]. Among various eliciting agents, we chose Bio-Gel polyacrylamide beads because these beads cannot be phagocytosed. Therefore, Bio-Gel-elicited macrophages are free of intracellular debris, making these cells suitable for studies pertaining to phagocytosis [22]. Using this agent we purified murine peritoneal macrophages which contained 8–10% mature macrophages and 60–70% immature macrophage (supplemental Fig. S6).

**Figure 4:**
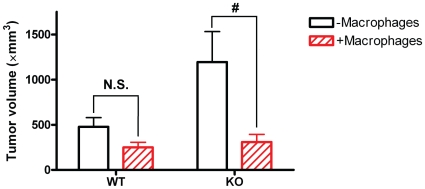
Macrophages can suppress tumor growth in adiponectin deficient mice. Tumor volume of B16F10 melanoma cells in adiponectin-knockout (KO) mice and wild-type (WT) control mice 14 days after the subcutaneous injection of B16F10 melanoma cells together with or without peritoneal macrophages (-macrophages: WT n = 7 and KO n = 8; +Macrophages: WT n = 8 and KO n = 8. #: p<0.05).

Next, we co-injected B16F10 cells and freshly isolated and characterized mouse peritoneal macrophages (supplemental Figure S6) subcutaneously into adiponectin knockout and wild-type mice. This would *mimic* the restoration of macrophage recruitment at the early stage of tumorigenesis. As shown in Figure 4, tumors grew in both adiponectin knockout and wild-type mice after co-injection of B16F10 cells and macrophages. However, tumor size after co-injection of macrophages was significantly reduced in adiponectin null mice (p<0.05) but not in wild-type mice. After 14 days post implantation, the average tumor volume was reduced by as much as 2–3 fold in adiponectin knockout mice, resulting in tumors similar in size to those growing in wild-type host animals (Figure 4, p<0.05).

## Discussion

To our knowledge the present study is the first that utilizes a syngeneic, immunocompetent mouse model to investigate the growth of transplanted tumor cells in an adiponectin deficient background. Our data clearly show that adiponectin plays a role in the suppression of the growth of implanted B16F10 melanoma and Lewis Lung Carcinoma tumors, most likely by enhancing the recruitment of macrophages to the tumor. We conclude that the increased tumor growth observed in adiponectin deficient mice is at least partly due to the reduction of macrophage infiltration and not by enhanced angiogenesis.

Recombinant adiponectin inhibits proliferation of breast cancer cell lines (MDA-MB-231 and MCF-7) in vitro[11], [12], suggesting that adiponectin may act as a cytokine which can directly suppress tumor growth in vivo. However, our data showed that recombinant adiponectin has no effect on the proliferation of cultured B16F10 cells, indicating that the inhibitory effects of adiponectin on cell proliferation are cell-type specific. This may be due to the distinct expression of the adiponectin receptor and/or different signal transduction pathways in various tissue types or even different cell lines of the same tissue type. There are three putative adiponectin receptors, adipoR1, adipoR2 [23] and T-cadherin [24] but it is unclear whether they can transmit adiponectin signals. AdipoR1 and AdipoR2 belong to the family of seven-trans-membrane spanning receptors with the N terminus intracellular, but the extracellular portion of these two receptors is small which is distinct from members of seven-spanning that bind peptide hormones. Although T-cadherin can bind adiponectin, it is likely not a signaling receptor since T-cadherin does not contain any trans-membrane domains. Thus, further identification of the functional adiponectin signaling receptor(s) should shed light on the role of adiponectin in glucose and fat metabolism and tumor biology.

A few recent reports addressed the growth of primary tumors in adiponectin knockout mice. For example, using a carcinogen induced colorectal cancer model, Nishihara et al. [25] showed that adiponectin knockout mice developed larger colorectal tumors. There are at least two possible explanations of this result. First, as indicated in the paper [25], tumor cells grew faster in adiponectin knockout mice. Second, the latency of colorectal tumors developed in adiponectin knockout mice might be shorter than in wild type mice. Thus, by 53 weeks after the carcinogen treatment, tumors in adiponectin knockout mice might exist longer and grow bigger than those developed in wild-type mice. However, this possibility was not discussed in the paper [25].

Primary MMTV-PyMT tumors grew faster in adiponectin haploinsufficient mice than in wild-type mice [26]. However, the tumor cells isolated from adiponectin haploinsufficient mice showed accelerated proliferation in culture, indicating that these cells are different from those isolated from wild-type mice. Paradoxically, utilizing the same cancer model, two other groups [7], [8] showed that adiponectin plays a pro-angiogenic role and may be pro-tumorigenic. Employing in vitro cell culture systems or in vivo administration of recombinant adiponectin or adenovirus that expresses adiponectin, several reports suggest that adiponectin may suppress the growth of other types of tumors [9], [12], [27], [28] although the underlying mechanisms may be different. Collectively, adiponectin is not only implicated in regulating glucose and fat metabolism, but also is capable of regulating the growth of multiple types of tumors, suggesting that adiponectin or its analogues [29], [30] may potentially serve as a novel anti-tumor agent.

In our study, adiponectin knockout mice exhibited normal numbers of circulating monocytes but markedly reduced infiltrating macrophages within the tumors. This indicates that adiponectin deficiency does not lead to monocyte defects, but instead impairs the recruitment of macrophages to tumor tissues. The mRNA expression of cytokines and growth factors, including MCP1(CCL2), TNFα, IL12, IFNγ, HGF, TGFβ1 and FasL, in tumors from adiponectin KO mice was similar to those from WT mice (data not shown). Thus, the underlying mechanisms still warrant further investigation. Strikingly, restoration of macrophages at the early stage of tumorigenesis by means of co-injection of macrophages completely reversed the increase in tumor growth in adiponectin null mice, while having little effect on tumor growth in wild-type recipients. This suggests that in the models used here adiponectin deficiency promotes tumor growth most likely by reducing macrophage infiltration. Our findings support the notion that macrophages can exert inhibitory effects on tumor growth at early stages of tumorigenesis, and provide an explanation for the association between low adiponectin levels and increased risk of many cancers. Future work is needed to dissect the precise roles and mechanisms of adiponectin in regulating tumorigenesis using various model systems; these experiments may help to shed light on the molecular basis through which obesity increases the risk for cancer formation and progression.

## Materials and Methods

### Mice

All animal experimental protocols were approved by the Committee on Animal Care of Massachusetts Institute of Technology (Protocol number: 1107-086-10). Adiponectin knockout mice were generously provided by Dr. Matsuzawa [31], and maintained in a C57BL/6J background.

### Cell lines and cell culture

Murine B16F10 melanoma and Lewis Lung Carcinoma cells were purchased from American Type Culture Collection (Manassas, Virginia). Human breast cancer cell line, MDA-MB-231, was obtained from the Weinberg Lab (Whitehead Institute for Biomedical Research, Cambridge, Massachusetts). All cell lines were maintained in DMEM supplemented with 10% fetal bovine serum, 2 mmole/L L-glutamine and penicillin-streptomycin, at 37°C in a humidified atmosphere of 5% CO_2_.

Murine peritoneal macrophages were isolated by p-100 Bio-Gel polyacrylamide beads (Bio-Rad laboratories, Hercules, California) elicitation [22]. Briefly, 1 ml sterile 2% (v/v) Bio-Gel suspension was injected into the peritoneal cavity of WT mice. 4 days later, elicited cells were recovered from the peritoneal fluids. Red blood cells were removed using RBC lysis buffer (eBioscience, San Diego, California), and the beads and other large particles were removed by filtering through the 20 µm sterile cell strainer.

### Mouse tumor cell transplantation

Six- to 8-week-old mice were injected subcutaneously on the back with 1×10^6^ B16F10 cells (with or without 2×10^6^ freshly isolated murine peritoneal macrophages), or 1×10^6^ Lewis Lung Carcinoma cells in 100 µl of DMEM containing 50% Matrigel (BD Biosciences, San Jose, California) [32]. Each group contains 5 to 10 mice. Tumor diameter was measured every 3 days, and tumor volume was calculated as (H×W^2^)/2.

### Experimental metastasis assay

Sub-confluent B16F10 melanoma cells were washed with PBS and detached by brief exposure to 0.25% trypsin and 0.2% EDTA. Cells were washed twice with PBS and resuspended in PBS at 1×10^6^ cells/ml. 100 µl mouse tumor cell suspension was injected to the lateral tail vein of mice. Ten days after the injection, lungs were harvested, and total numbers of metastatic nodules in the lung of individual mice were counted under a dissecting microscope.

### Immunohistochemistry

Tumors were harvested from CO_2_-euthanized mice and fixed in 10% neutral buffered formalin. 4-5 micrometer paraffin-embedded sections of tumors were used for immunohistochemistry. Immunohistochemical staining of tumor sections against antibodies anti-cleaved caspase 3, anti-Ki-67, anti-MECA-32 and anti-F4/80 was performed by the core facility of the Division of Comparative Medicine (Massachusetts Institute of Technology, Cambridge, Massachusetts).

Vessel density in tumors was determined by anti-MECA-32 IHC staining. Briefly, any MECA-32+ endothelial cell or cell cluster that was separated from adjacent micro-vessels was counted as one vessel. The vessel density was expressed as the absolute number of micro-vessels per field (100× field).

Intra-tumor macrophage infiltration was quantified by anti-F4/80 IHC staining. Any F4/80+ stained mononuclear cell was counted as one macrophage. The macrophage infiltration was expressed as the absolute number of macrophages per field (200× field). 3 fields/section from non-necrotic regions in 3–8 tumors were counted in a blinded fashion.

### Flow cytometry

FITC-conjugated anti-F4/80 and PE-conjugated anti-CD11b (eBioscience, San Diego, California) antibodies were used to determine the purity of peritoneal monocytes. FITC-conjugated anti-CD19, PE-conjugated anti-CD11b, APC-conjugated anti-CD3 and PE-cy7-conjugated anti-Gr-1 (eBioscience, San Diego, California) were used to stain B cells, T cells, monocytes and granulocytes present in the blood of mice. Fractions of apoptotic cells in transplanted B16F10 tumors were determined by FACS using an Annexin V: PE Apoptosis Detection Kit (BD Biosciences, San Jose, California).

### Statistical analysis

Data are presented as mean ± standard error and by unpaired two-tailed Student's *t* test. *P* values of <0.05 were regarded as statistically significant.

## Supporting Information

Figure S1 Recombinant adiponectin does not affect B16F10 cell proliferation. 50,000 cells per well are seeded into 6-well plate in the presence or absence of 30 ug/ml recombinant adiponectin. Cell numbers were counted at 24 hours or 48 hours (B16F10: 4 replicates per group; MDA-MB-231: 3 replicates per group. *: p<0.05). Mouse recombinant adiponectin was expressed in HEK293 cells.
(0.17 MB TIF)

Figure S2 LLC tumors from adiponectin knockout (KO) mice display similar apoptosis, mitosis and angiogenesis compared with those from wild type (WT) mice. A. Immunohistochemistry staining of LLC tumor sections from wild type and adiponectin knockout mice. Antibodies: cleaved caspase 3, Ki-67 and MECA-32 (vessels are brown and indicated by arrows. scale bar, 50 um). B. Percentage of mitotic cells does not change in tumors from adiponectin null mice (Fields counted: WT, n = 4; KO, n = 4. p>0.05). C. Adiponectin deficiency does not affect vessel density in transplanted tumors (Fields counted: WT, n = 7; KO, n = 10. p>0.05).
(3.59 MB TIF)

Figure S3 FACS analysis of apoptosis in B16F10 tumors from adiponectin null (KO) and wild type (WT) mice. B16F10 tumors were harvested 9 days after cancer cell implantation. Then, B16F10 cells were resuspended by pipetting up and down, and strained through the 70 um cell drainer. The percentage of apoptotic cells in transplanted B16F10 tumors were determined by FACS using an Annexin V : PE Apoptosis Detection Kit (BD Biosciences, San Jose, California).
(0.10 MB TIF)

Figure S4 Macrophage infiltration is reduced in LLC tumors from adiponectin knockout (KO) mice. A. Immunohistochemistry staining of LLC tumor sections from wild type (WT) and adiponectin knockout (KO) mice against an anti-macrophage antibody F4/80. A lower infiltration of macrophages (brown, indicated by black arrows; red arrows indicate the macrophage infiltration on the edge of tumors) was observed in tumors grown in adiponectin null mice. (Scale bar, A: 200 um, B: 50 um). B. Microscopic counts indicate that adiponectin deficiency decreases intra-tumor macrophage infiltration (Fields counted: WT, n = 9; KO n = 9; p<0.05).
(4.37 MB TIF)

Figure S5 Adiponectin deficiency does not alter monocyte (CD11b+Gr-1-), granulocyte (CD11b+Gr-1+), T cells (CD3+) and B cell (CD19+) number in peripheral blood. (adiponectin knockout mice (KO), n = 11; wild type mice (WT), n = 9. p>0.05).
(0.17 MB TIF)

Figure S6 FACS analysis of the purity of peritoneal macrophages. (Macrophage, F4/80+CD11b+; immature macrophage, F4/80-CD11b+, n = 4).
(0.30 MB TIF)

## References

[1] Li S, Shin HJ, Ding EL, van Dam RM (2009). Adiponectin levels and risk of type 2 diabetes: a systematic review and meta-analysis.. Jama.

[2] Antoniades C, Antonopoulos AS, Tousoulis D, Stefanadis C (2009). Adiponectin: from obesity to cardiovascular disease.. Obes Rev.

[3] Matsuzawa Y, Funahashi T, Kihara S, Shimomura I (2004). Adiponectin and metabolic syndrome.. Arterioscler Thromb Vasc Biol.

[4] Kelesidis I, Kelesidis T, Mantzoros CS (2006). Adiponectin and cancer: a systematic review.. Br J Cancer.

[5] Ouchi N, Kobayashi H, Kihara S, Kumada M, Sato K (2004). Adiponectin stimulates angiogenesis by promoting cross-talk between AMP-activated protein kinase and Akt signaling in endothelial cells.. J Biol Chem.

[6] Shibata R, Ouchi N, Kihara S, Sato K, Funahashi T (2004). Adiponectin stimulates angiogenesis in response to tissue ischemia through stimulation of amp-activated protein kinase signaling.. J Biol Chem.

[7] Denzel MS, Hebbard LW, Shostak G, Shapiro L, Cardiff RD (2009). Adiponectin deficiency limits tumor vascularization in the MMTV-PyV-mT mouse model of mammary cancer.. Clin Cancer Res.

[8] Landskroner-Eiger S, Qian B, Muise ES, Nawrocki AR, Berger JP (2009). Proangiogenic contribution of adiponectin toward mammary tumor growth in vivo.. Clin Cancer Res.

[9] Brakenhielm E, Veitonmaki N, Cao R, Kihara S, Matsuzawa Y (2004). Adiponectin-induced antiangiogenesis and antitumor activity involve caspase-mediated endothelial cell apoptosis.. Proc Natl Acad Sci U S A.

[10] Man K, Ng KT, Xu A, Cheng Q, Lo CM (2010). Suppression of Liver Tumor Growth and Metastasis by Adiponectin in Nude Mice through Inhibition of Tumor Angiogenesis and Downregulation of Rho Kinase/IFN-Inducible Protein 10/Matrix Metalloproteinase 9 Signaling.. Clin Cancer Res.

[11] Kang JH, Lee YY, Yu BY, Yang BS, Cho KH (2005). Adiponectin induces growth arrest and apoptosis of MDA-MB-231 breast cancer cell.. Arch Pharm Res.

[12] Arditi JD, Venihaki M, Karalis KP, Chrousos GP (2007). Antiproliferative effect of adiponectin on MCF7 breast cancer cells: a potential hormonal link between obesity and cancer.. Horm Metab Res.

[13] Park PH, McMullen MR, Huang H, Thakur V, Nagy LE (2007). Short-term treatment of RAW264.7 macrophages with adiponectin increases tumor necrosis factor-alpha (TNF-alpha) expression via ERK1/2 activation and Egr-1 expression: role of TNF-alpha in adiponectin-stimulated interleukin-10 production.. J Biol Chem.

[14] Folco EJ, Rocha VZ, Lopez-Ilasaca M, Libby P (2009). Adiponectin inhibits pro-inflammatory signaling in human macrophages independent of interleukin-10.. J Biol Chem.

[15] Takemura Y, Ouchi N, Shibata R, Aprahamian T, Kirber MT (2007). Adiponectin modulates inflammatory reactions via calreticulin receptor-dependent clearance of early apoptotic bodies.. J Clin Invest.

[16] Ohashi K, Parker JL, Ouchi N, Higuchi A, Vita JA Adiponectin promotes macrophage polarization toward an anti-inflammatory phenotype..

[17] Sica A, Allavena P, Mantovani A (2008). Cancer related inflammation: the macrophage connection.. Cancer Lett.

[18] Fiumara A, Belfiore A, Russo G, Salomone E, Santonocito GM (1997). In situ evidence of neoplastic cell phagocytosis by macrophages in papillary thyroid cancer.. J Clin Endocrinol Metab.

[19] Bonnotte B, Larmonier N, Favre N, Fromentin A, Moutet M (2001). Identification of tumor-infiltrating macrophages as the killers of tumor cells after immunization in a rat model system.. J Immunol.

[20] Kumar R, Yoneda J, Fidler IJ, Dong Z (1999). GM-CSF-transduced B16 melanoma cells are highly susceptible to lysis by normal murine macrophages and poorly tumorigenic in immune-compromised mice.. J Leukoc Biol.

[21] Lamagna C, Aurrand-Lions M, Imhof BA (2006). Dual role of macrophages in tumor growth and angiogenesis.. J Leukoc Biol.

[22] Zhang X, Goncalves R, Mosser DM (2008). The isolation and characterization of murine macrophages.. Curr Protoc Immunol Chapter 14: Unit 14.

[23] Yamauchi T, Kamon J, Ito Y, Tsuchida A, Yokomizo T (2003). Cloning of adiponectin receptors that mediate antidiabetic metabolic effects.. Nature.

[24] Hug C, Wang J, Ahmad NS, Bogan JS, Tsao TS (2004). T-cadherin is a receptor for hexameric and high-molecular-weight forms of Acrp30/adiponectin.. Proc Natl Acad Sci U S A.

[25] Nishihara T, Baba M, Matsuda M, Inoue M, Nishizawa Y (2008). Adiponectin deficiency enhances colorectal carcinogenesis and liver tumor formation induced by azoxymethane in mice.. World J Gastroenterol.

[26] Lam JB, Chow KH, Xu A, Lam KS, Liu J (2009). Adiponectin haploinsufficiency promotes mammary tumor development in MMTV-PyVT mice by modulation of phosphatase and tensin homolog activities.. PLoS One.

[27] Dieudonne MN, Bussiere M, Dos Santos E, Leneveu MC, Giudicelli Y (2006). Adiponectin mediates antiproliferative and apoptotic responses in human MCF7 breast cancer cells.. Biochem Biophys Res Commun.

[28] Wang Y, Lam JB, Lam KS, Liu J, Lam MC (2006). Adiponectin modulates the glycogen synthase kinase-3beta/beta-catenin signaling pathway and attenuates mammary tumorigenesis of MDA-MB-231 cells in nude mice.. Cancer Res.

[29] Wong GW, Krawczyk SA, Kitidis-Mitrokostas C, Ge G, Spooner E (2009). Identification and characterization of CTRP9, a novel secreted glycoprotein, from adipose tissue that reduces serum glucose in mice and forms heterotrimers with adiponectin.. Faseb J.

[30] Wong GW, Krawczyk SA, Kitidis-Mitrokostas C, Revett T, Gimeno R (2008). Molecular, biochemical and functional characterizations of C1q/TNF family members: adipose-tissue-selective expression patterns, regulation by PPAR-gamma agonist, cysteine-mediated oligomerizations, combinatorial associations and metabolic functions.. Biochem J.

[31] Maeda N, Shimomura I, Kishida K, Nishizawa H, Matsuda M (2002). Diet-induced insulin resistance in mice lacking adiponectin/ACRP30.. Nat Med.

[32] Ho-Tin-Noe B, Goerge T, Cifuni SM, Duerschmied D, Wagner DD (2008). Platelet granule secretion continuously prevents intratumor hemorrhage.. Cancer Res.

