# Supplementary figures and images for: Adiponectin Deficiency Promotes Tumor Growth in Mice by Reducing Macrophage Infiltration
Source: PLoS One. 2010 Aug 5;5(8):e11987. doi: 10.1371/journal.pone.0011987 (PMC2916827; doi:10.1371/journal.pone.0011987)

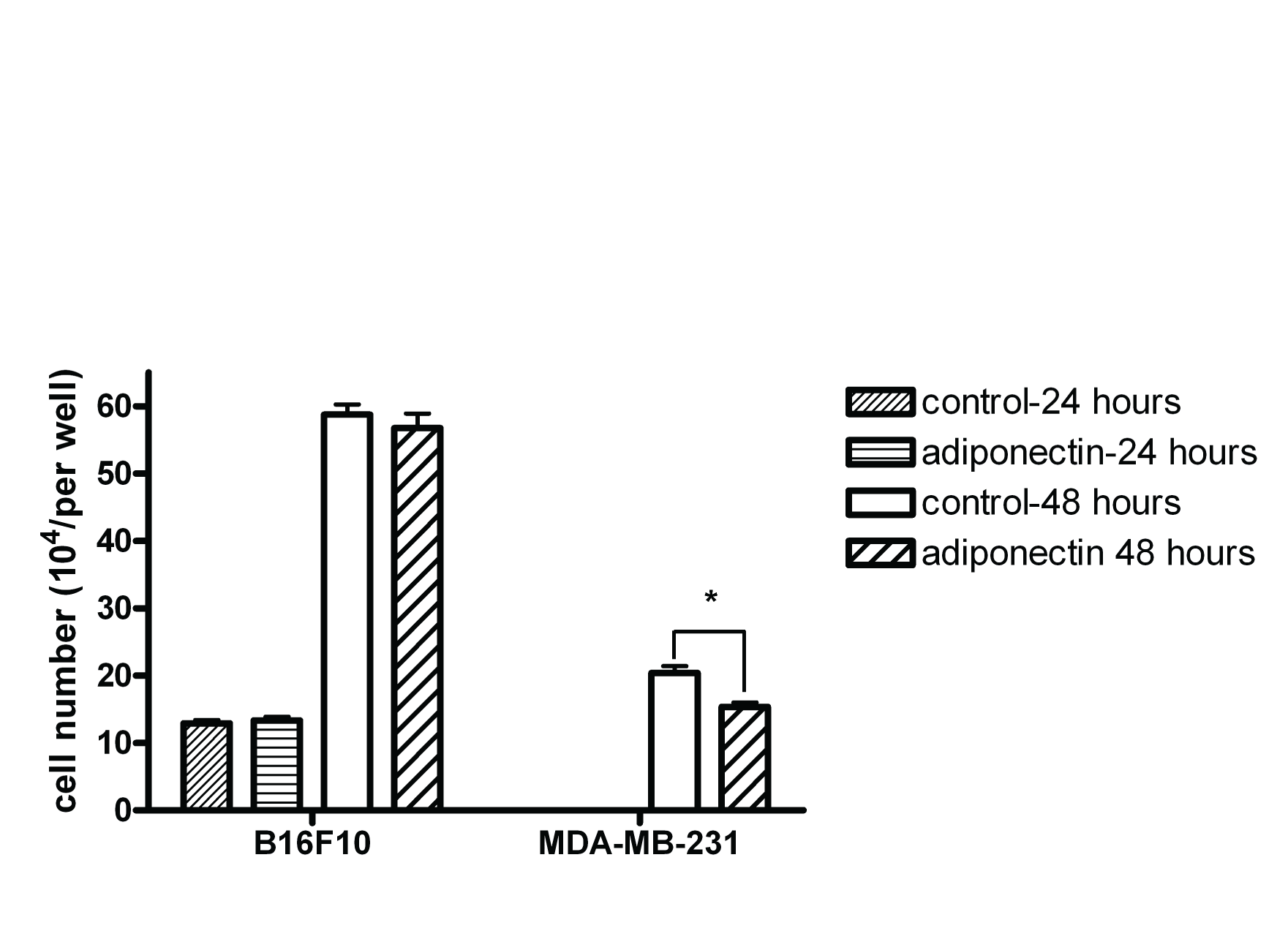

Supplement: Figure S1 — Recombinant adiponectin does not affect B16F10 cell proliferation. 50,000 cells per well are seeded into 6-well plate in the presence or absence of 30 ug/ml recombinant adiponectin. Cell numbers were counted at 24 hours or 48 hours (B16F10: 4 replicates per group; MDA-MB-231: 3 replicates per group. *: p<0.05). Mouse recombinant adiponectin was expressed in HEK293 cells. (0.17 MB TIF) [file pone.0011987.s001.tif]

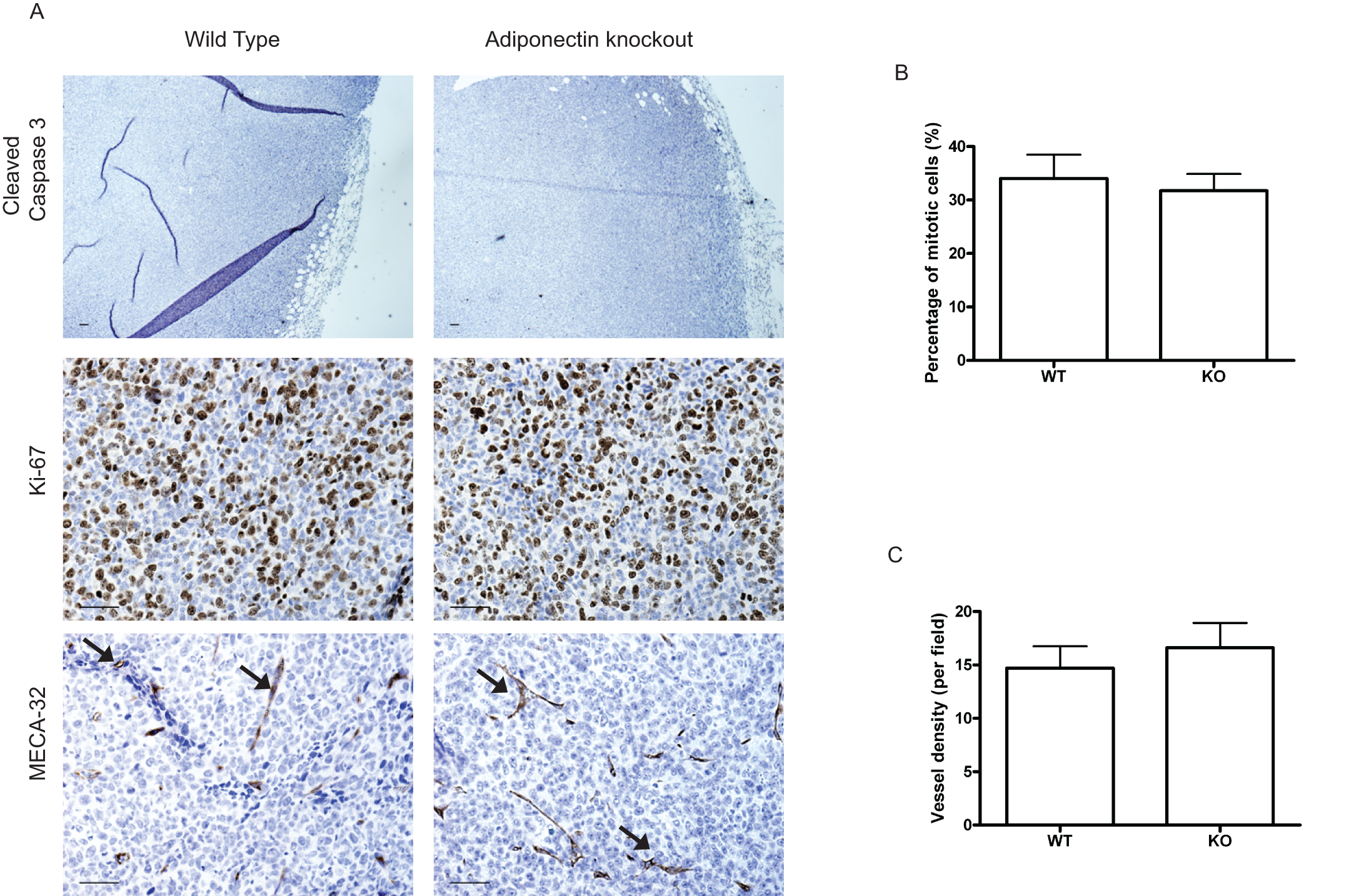

Supplement: Figure S2 — LLC tumors from adiponectin knockout (KO) mice display similar apoptosis, mitosis and angiogenesis compared with those from wild type (WT) mice. A. Immunohistochemistry staining of LLC tumor sections from wild type and adiponectin knockout mice. Antibodies: cleaved caspase 3, Ki-67 and MECA-32 (vessels are brown and indicated by arrows. scale bar, 50 um). B. Percentage of mitotic cells does not change in tumors from adiponectin null mice (Fields counted: WT, n = 4; KO, n = 4. p>0.05). C. Adiponectin deficiency does not affect vessel density in transplanted tumors (Fields counted: WT, n = 7; KO, n = 10. p>0.05). (3.59 MB TIF) [file pone.0011987.s002.tif]

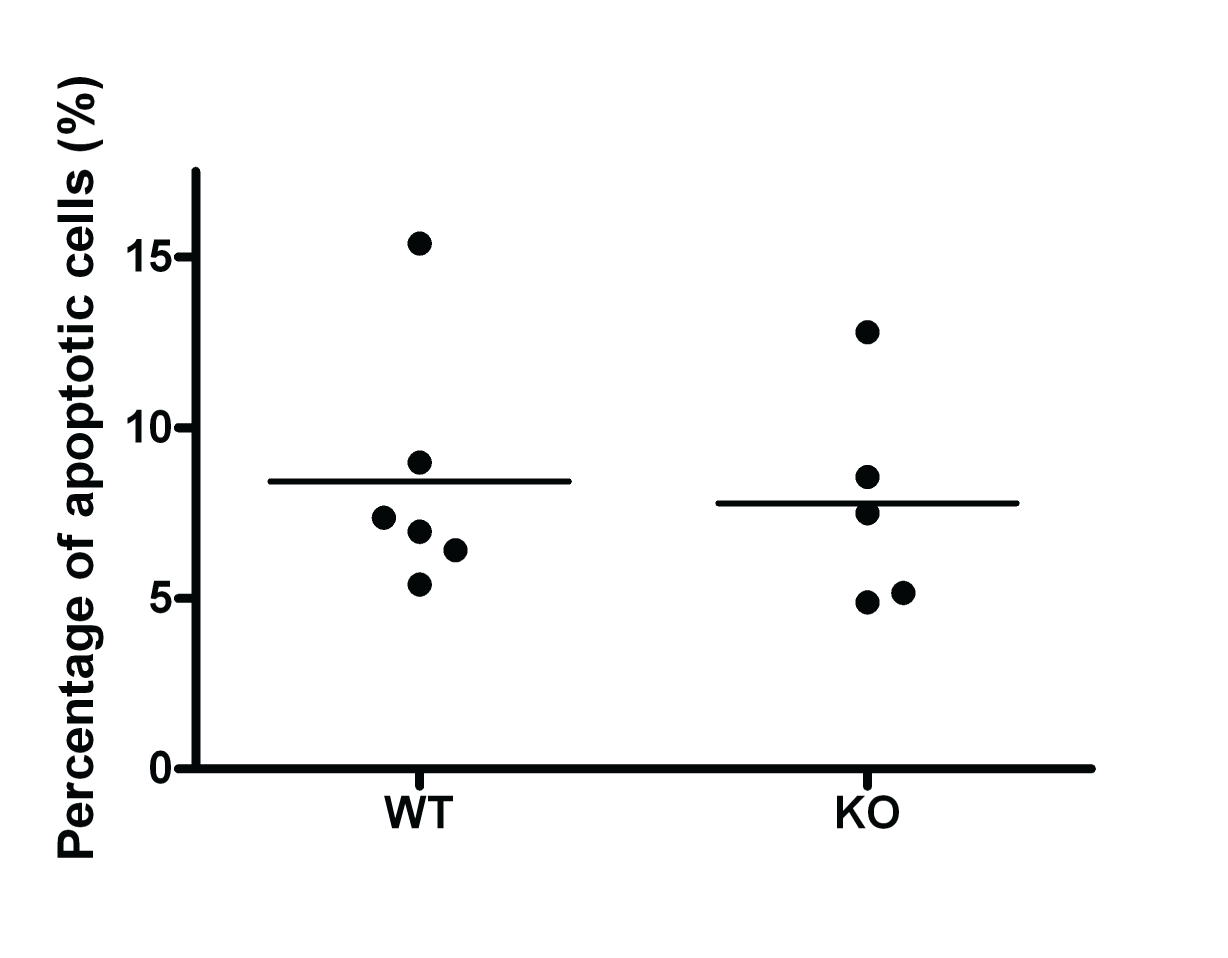

Supplement: Figure S3 — FACS analysis of apoptosis in B16F10 tumors from adiponectin null (KO) and wild type (WT) mice. B16F10 tumors were harvested 9 days after cancer cell implantation. Then, B16F10 cells were resuspended by pipetting up and down, and strained through the 70 um cell drainer. The percentage of apoptotic cells in transplanted B16F10 tumors were determined by FACS using an Annexin V : PE Apoptosis Detection Kit (BD Biosciences, San Jose, California). (0.10 MB TIF) [file pone.0011987.s003.tif]

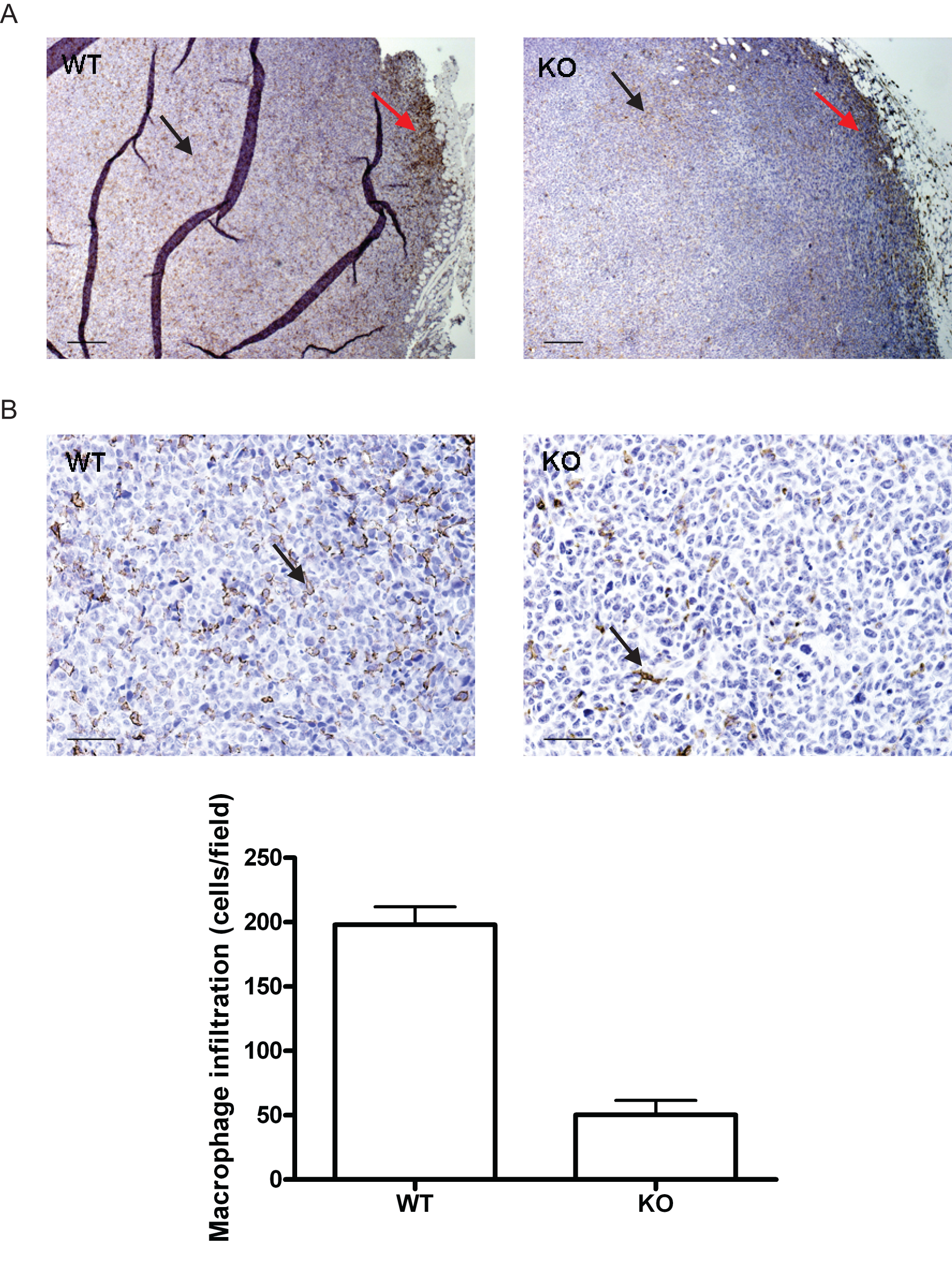

Supplement: Figure S4 — Macrophage infiltration is reduced in LLC tumors from adiponectin knockout (KO) mice. A. Immunohistochemistry staining of LLC tumor sections from wild type (WT) and adiponectin knockout (KO) mice against an anti-macrophage antibody F4/80. A lower infiltration of macrophages (brown, indicated by black arrows; red arrows indicate the macrophage infiltration on the edge of tumors) was observed in tumors grown in adiponectin null mice. (Scale bar, A: 200 um, B: 50 um). B. Microscopic counts indicate that adiponectin deficiency decreases intra-tumor macrophage infiltration (Fields counted: WT, n = 9; KO n = 9; p<0.05). (4.37 MB TIF) [file pone.0011987.s004.tif]

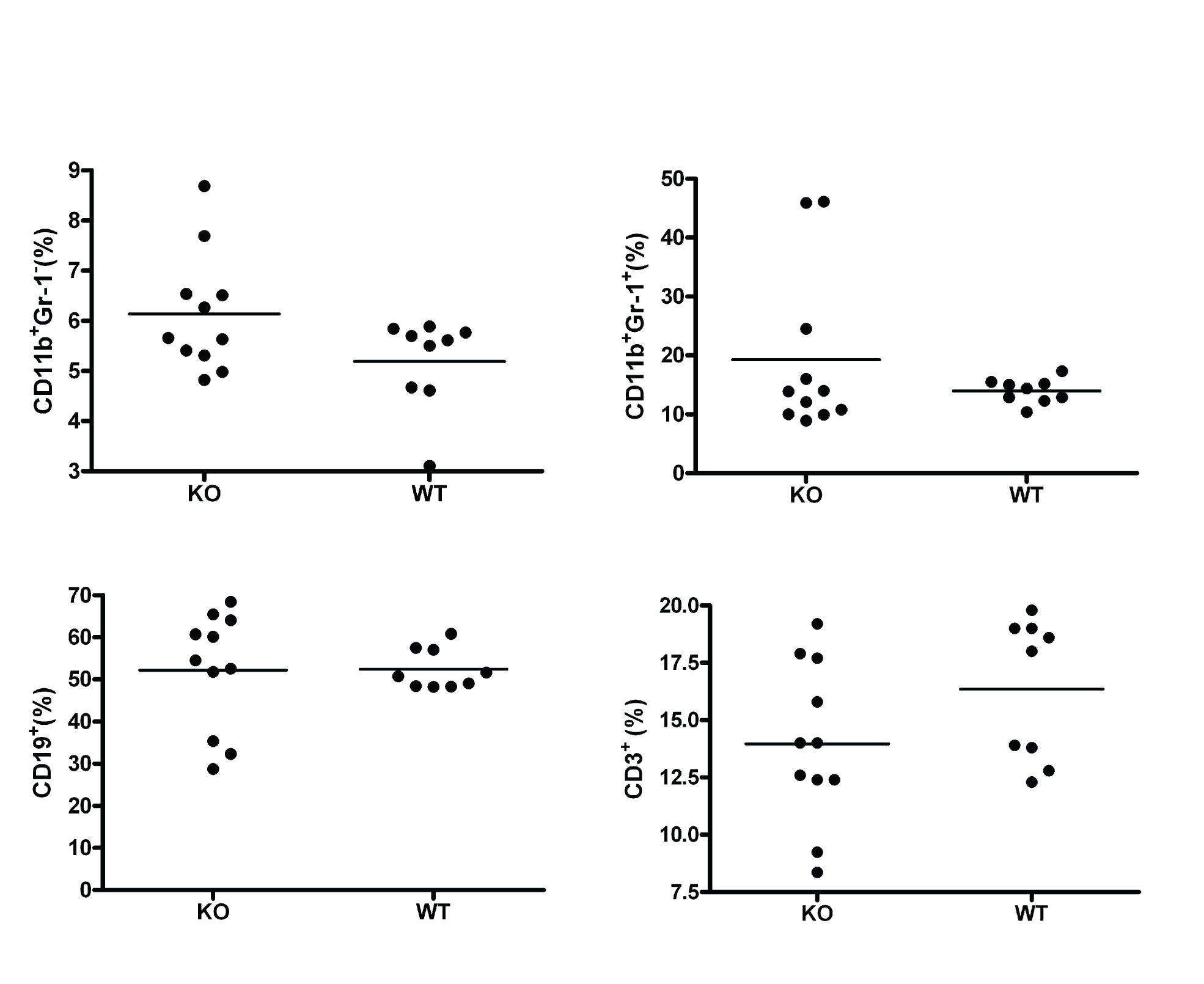

Supplement: Figure S5 — Adiponectin deficiency does not alter monocyte (CD11b+Gr-1-), granulocyte (CD11b+Gr-1+), T cells (CD3+) and B cell (CD19+) number in peripheral blood. (adiponectin knockout mice (KO), n = 11; wild type mice (WT), n = 9. p>0.05). (0.17 MB TIF) [file pone.0011987.s005.tif]

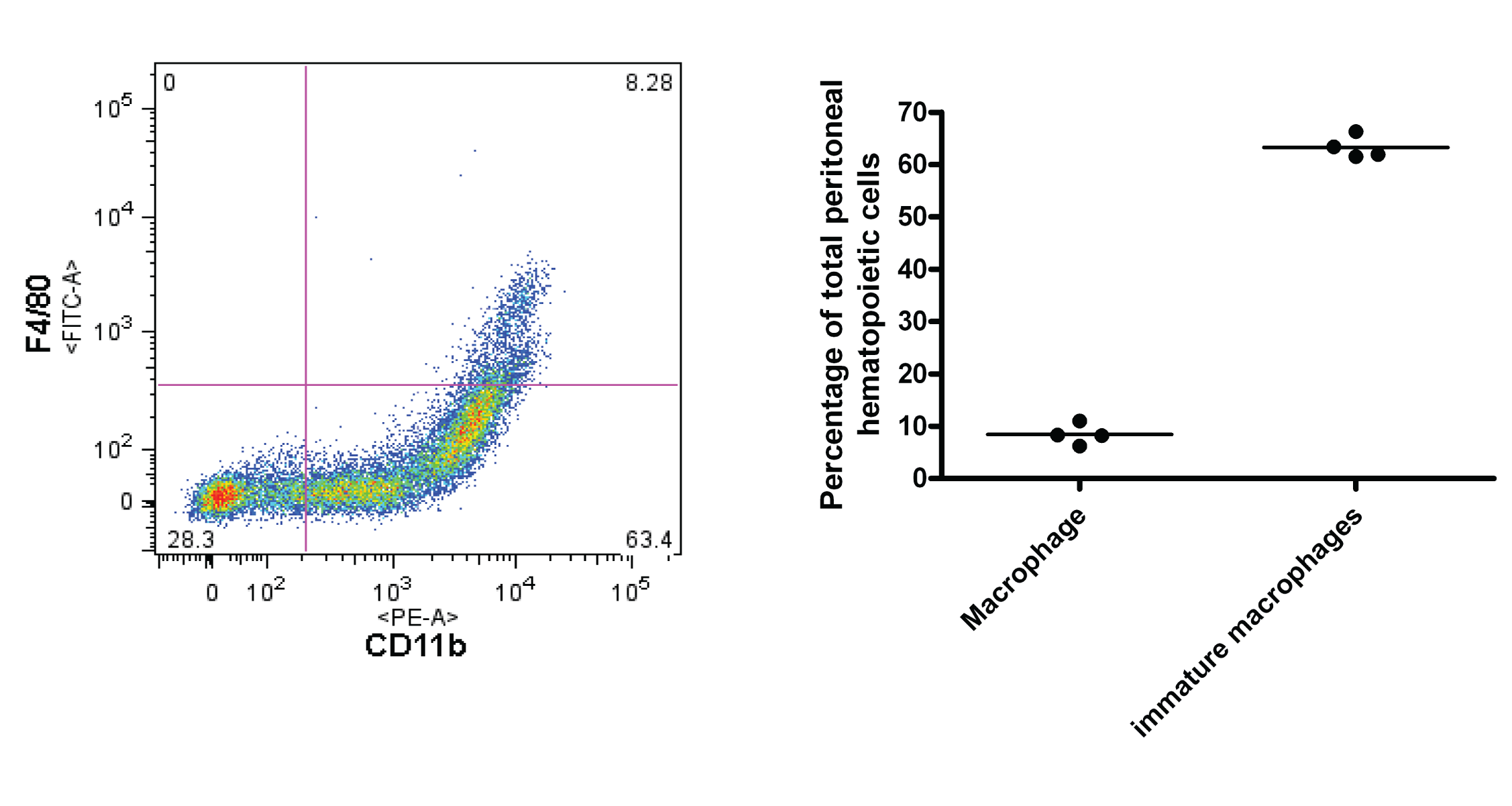

Supplement: Figure S6 — FACS analysis of the purity of peritoneal macrophages. (Macrophage, F4/80+CD11b+; immature macrophage, F4/80-CD11b+, n = 4). (0.30 MB TIF) [file pone.0011987.s006.tif]
